# Notes and Notices

**Published:** 1890-09

**Authors:** 


### NOTES AND NOTICES.

REMOVALS.—Dr. James W. Ward desires to announce his removal, after July 20th, to 924 Geary Street, between Larkin and Polk. Office hours: 10 to 12 A. M., 7 to 7.30 P. M. Telephone 2123.

Drs. Bœricke & Dewey have removed to 824 Sutter Street, San Francisco, five doors below their old office. Telephone 2207.

Dr. G. E. Gramm has removed from Philadelphia to Ardmore, Montgomery County, Pennsylvania.

Dr. Horace P. Holmes, formerly of Sycamore, Illinois, has associated himself with Dr. C. M. Dinsmoor as partner, and will hereafter practice medicine at Rooms Nos. 30 and 31 Douglas Block, Omaha, Nebraska.

